# Co-Rumination with Peers Predicts Risk for Depression in Offspring of Mothers with Major Depressive Disorder

**DOI:** 10.1007/s10802-026-01486-3

**Published:** 2026-07-16

**Authors:** Brianna M. Lind, Brandon E. Gibb

**Affiliations:** Department of Psychology, Binghamton University (SUNY), Binghamton, NY 13902-6000 USA

**Keywords:** Co-rumination, Major depression, Intergenerational transmission, Child

## Abstract

Although offspring of mothers with major depressive disorder (MDD) are at heightened risk for the disorder, most never develop depression, highlighting the need to identify moderators of risk. Co-rumination, the tendency to frequently and passively discuss problems with a peer without any active problem-solving, is a known risk factor in youth and may help to identify which offspring of mothers with MDD are at greatest risk. We tested this hypothesis using data from a multi-wave longitudinal study, examining prospective changes in youths’ depressive symptoms and risk for MDD onset during the two-year follow-up. Participants were 214 children (ages 8–14 at baseline) of mothers with (*n* = 106) or without (*n* = 108) a history of MDD. At baseline, mothers completed a diagnostic interview, and children completed a self-report measure of co-rumination. Follow up assessments, completed every 6 months for 2 years, were used to assess depressive symptoms and the onset of MDD episodes in children. Supporting our hypothesis, among offspring of mothers with MDD, but not among offspring of never depressed mothers, higher levels of co-rumination at the baseline assessment predicted higher levels of depressive symptoms in offspring at baseline the assessment that were maintained through the end of the follow-up period, as well as increased risk for the onset of MDD across the follow-up. These results highlight the role of co-rumination, which is a modifiable risk factor that may be targeted in prevention and early intervention programs to reduce risk for the intergenerational transmission of depression.

Rates of depression increase dramatically from childhood to adolescence (Avenevoli et al., 2008; Mojtabai et al., 2016) and the prevalence of depression in youth has increased significantly over the past decade (Daly, 2022; Shorey et al., 2022). One of the strongest risk factors for major depressive disorder (MDD) in youth is a family history of the disorder (Goodman, 2020). Indeed, offspring of mothers with MDD are three to six times more likely to develop MDD themselves than offspring of never depressed mothers (Gotlib et al., 2020). However, most offspring of mothers with MDD never develop depression, suggesting the presence of important moderating variables. Mechanisms of risk for the intergenerational transmission include both genetic and environmental influences (Goodman, 2020). For example, there is growing evidence that offspring of mothers with a history of MDD during their lives experience higher levels of stress both at home and with their peers compared to offspring of never depressed mothers (Adrian & Hammen, 1993; Feurer et al., 2016). Although stress within the home may decrease once the mothers’ depression remits, difficulties within peer relations remain and appear more chronic (Feurer et al., 2016). Individual differences in how youth cope with this stress may help to identify those at highest risk.

When facing stressors, youth often turn to peers and close friends for support (for a review, see Schwartz-Mette et al., 2020). Although parents play a central role in shaping children’s emotional development and risk for psychopathology, peers become increasingly important across late childhood and adolescence as youth spend more time with friends, develop greater autonomy from parents, and turn to peers for emotional support and help coping with stress (Donlan et al., 2015; Licitra-Kleckler & Waas, 1993). This developmental shift does not replace parental influence but rather results in youth being embedded simultaneously within family and peer interpersonal environments that jointly contribute to depression risk (Auerbach et al., 2011; Hammen, 2009).

Social support from peers is a known protective factor for depression in children and adolescents (Cairns et al., 2014; Rueger et al., 2016). However, not all forms of social support are equally beneficial or lead to positive outcomes, as some friendships are characterized by dysfunctional relationship styles that promote maladaptive behaviors (Dishion & Tipsord, 2011). Co-rumination, which can be considered an interpersonal form of rumination, refers to the tendency to frequently and passively discuss and rehash problems with a peer, without any active problem-solving and is most commonly evaluated within the context of the youth’s closest same-sex relationship (Rose, 2002). Although co-rumination can have some interpersonal benefits (e.g., increases in perceived friendship quality; Rose, 2002, 2021; Rose et al., 2007), there is growing evidence that it also increases risk for depressive symptoms and diagnoses in youth. Specifically, co-rumination is associated with current depressive symptoms in youth (for review, see Spendelow et al., 2017; Tilton-Weaver & Rose, 2023) and predicts prospective increases in youths’ levels of depression over time (Hankin et al., 2010; Stone et al., 2011). Moreover, higher levels of co-rumination predict an increased risk for the development of depressive episodes (Stone et al., 2011). Therefore, co-rumination appears to be an important risk factor in youth, the use of which may be maintained by increases in perceived friendship quality even though it also increases youth risk for depression.

Although co-rumination appears to be an important interpersonal risk factor for depression in youth generally, prior research suggests that its effects may be particularly pronounced among adolescents already exposed to elevated vulnerability or interpersonal stress. Specifically, evidence suggests that co-rumination but may amplify existing vulnerabilities by exacerbating responses to interpersonal stress. For example, co-rumination has been shown to strengthen associations between interpersonal stress and depressive symptoms (Bastin et al., 2015), exacerbate stress generation among adolescents with depressive symptoms, particularly in peer contexts (Rose et al., 2017), and intensify the psychological effects of peer victimization (Huang et al., 2022). These findings suggest that co-rumination may increase the salience of interpersonal stressors and reinforce maladaptive cognitive and emotional responses to stress, thereby amplifying rather than merely adding to pre-existing vulnerabilities. Thus, among offspring of mothers with MDD, who are already exposed to elevated levels of interpersonal stress both inside and outside the home (Adrian & Hammen, 1993; Feurer et al., 2016), co-rumination may exacerbate existing vulnerability and contribute to greater depression risk.

Importantly, co-rumination is associated with other established risk factors for youth depression, including individual rumination (Piraman et al., 2016; Badawi & Ingram, 2025), though research suggests that co-rumination represents a distinct interpersonal risk process for depression. Indeed, recent research suggests that co-rumination predicts depression risk above and beyond broader personality traits, including neuroticism, supporting its incremental validity as a distinct interpersonal vulnerability factor (Luo & Luo, 2025). Whereas rumination is an intrapersonal cognitive process, co-rumination is a dyadic interpersonal process that may be reinforced by a relationship partner. Moreover, prior work suggests that co-rumination and rumination are related but separable constructs with distinct interpersonal consequences (Calmes & Roberts, 2008; Spendelow et al., 2017). Consistent with this distinction, Rose et al. (2017) found that co-rumination exacerbated stress generation effects associated with adolescent depression, and these effects remained significant when statistically controlling for the effects of rumination. Similarly, research using observational behavioral methods suggests that aspects of co-rumination characterized by dwelling on negative affect remain associated with depressive symptoms even after accounting for the role of rumination (Rose et al., 2014). Thus, co-rumination appears to play a unique role in depression risk.

The goal of this study was to determine whether this established risk factor for depression in youth may help to identify which offspring of mothers with a history of MDD are at greatest risk for depression themselves. Consistent with a “two hit” model of risk for depression, we predicted that offspring of mothers with a history of MDD during their lives who also exhibit high levels of co-rumination with their peers would be at greatest risk for depression. In testing this hypothesis, we focused on youths’ trajectories of depressive symptoms, as well as risk for MDD onset, over a two-year follow-up. We focused on youth’s levels of co-rumination with their best, same-sex peer because we were specifically interested in determining how familial and peer-related processes may combine to increase risk for depression. In addition, decades of research have established co-rumination with same-sex peers as a risk factor for youth depression (for reviews, see Badawi & Ingram, 2025; Dong et al., 2025; Rose, 2021) and it appears to be a stronger predictor of depression risk than co-rumination with others, including mothers (e.g., Calmes & Roberts, 2008; Waller & Rose, 2013). Finally, we focused on youth aged 8–14 during at the baseline assessment (aged 10–16 at the end of the study) because this is a developmental window of heightened risk for depression and a period during which feedback and support from peers increases in importance (Avenevoli et al., 2015; Brown & Larson, 2009; Steinberg & Morris, 2001).

In addition to our primary analyses, we conducted exploratory analyses to determine whether any of the relations were moderated by youths’ age or sex. As noted above, the transition from late childhood to adolescence is marked by significant increases in depression risk (Daly, 2022; Hankin et al., 1998). It is also characterized by the increased salience of peer relationships (Brown & Larson, 2009; Steinberg & Morris, 2001), which is accompanied by age-based increases in co-rumination (DiGiovanni et al., 2022; Felton et al., 2019; Stone et al., 2011). With regard to the potential moderating role of youth sex, there is evidence that risk for the intergenerational transmission of depression may be higher among daughters than sons of mothers with MDD (Goodman, 2020). This said, previous research has found little evidence of age- or sex-based differences in the link between co-rumination and depression risk in youth (Badawi & Ingram, 2025; Dong et al., 2025; Spendelow et al., 2017). Given this, we did not make any specific hypotheses for these analyses.

## Method

### Participants

Participants in this study were 214 mother-offspring dyads recruited from the community between 2010 and 2014 as part of a larger study examining risk for the intergenerational transmission of depression. To qualify for the study, mothers were required to either have a history of MDD during the child’s lifetime (*n* = 106) according to the DSM-IV (American Psychiatric Association, 1994) or have no lifetime history of any depressive disorder (*n* = 108).^1^ Mothers were classified as having a history of MDD if they met criteria for at least one depressive episode during their child’s lifetime, regardless of the developmental timing of the episode. Exclusion criteria for both groups included symptoms of schizophrenia, alcohol or substance dependence within the last 6 months, or a history of bipolar disorder. Offspring had to be between the ages 8- to 14-year-old at the initial assessment and be the biological child of the participating mother. Exclusion criteria for the youth were that they could not have a developmental or learning disability per mother report. Moreover, only one child per family could participate in the study. When more than one child was eligible for participation, one child was chosen at random for participation.

The average age for youth in this study was 11.51 years (*SD* = 1.90) at baseline and 51.40% were female. Of the youth, 82.24% were White, 4.67% were Black, 10.28% were multiracial, and the remainder were from other groups. The average age of the mothers in our sample was 40.70 years (*SD* = 6.95). Of the mothers, 88.79% were White, 4.21% were Black, 3.27% were multiracial, and the remainder were from other groups. The median annual family income was $50,000-$55,000.

### Measures

Mothers were administered the Structured Clinical Interview for DSM-IV Axis I Disorders (SCID-I; First et al., 2002) at the baseline assessment to determine inclusion/exclusion criteria. The SCID-I is a well-validated structured diagnostic interview for adults (First et al., 2002). As noted above, 106 mothers met criteria for at least one episode of an MDD diagnosis during their child’s lifetime and the remaining 108 had no lifetime history of any depressive disorder. To assess inter-rater reliability of diagnoses, a random subset of 20 SCID-I interviews from this project were selected and coded by a second interviewer The inter-rater reliability for diagnoses of MDD in mothers was excellent (κ = 1.00).

Diagnoses of MDD in offspring were assessed using the Schedule for Affective Disorders and Schizophrenia for School-Age Children – Present and Lifetime Version (K-SADS-PL; Kaufman et al., 1997). The K-SADS-PL is a widely used, developmentally sensitive interview validated for use in children and adolescents (Kaufman et al., 1997). Following standard practice, the K-SADS-PL was administered to mothers and children separately to assess for diagnoses of MDD in the offspring. The same person administered the K-SADS-PL to mothers and children, and a different person administered the SCID-I to assess mothers’ MDD. At each follow-up assessment, the K-SADS-PL was used to determine whether children met criteria for MDD during the follow-up interval and, if so, the date of MDD onset. None of the children in this study met criteria for current MDD at the baseline assessment and 16 children experienced an onset of at least one episode of MDD during the two-year follow-up (11 of whom had a mother with MDD). As with the SCID-I, a random subset of 20 K-SADS-PL interviews from this project were selected and coded by a second interviewer, and the inter-rater reliability for diagnoses of MDD in children was excellent (κs = 1.00).

At each assessment, youths’ current levels of depressive symptoms were assessed using the Children’s Depression Rating Scale-Revised (CDRS-R; Poznanski & Mokros, 1996). The CDRS-R is a 17-item rating scale that is interviewer-administered measure. Depressive symptoms are rated on a 5-point scale for three items (e.g., appetite disturbance) and on a 7-point scale for 14 items (e.g., irritability). The total score (sum of the 17 items) was used in the analyses, with higher scores indicating greater depressive symptom severity. The CDRS-R has demonstrated excellent reliability and validity in child and adolescent samples (e.g., Mayes et al., 2010; Poznanski & Mokros, 1996) and it exhibited good internal consistency across all five assessments in this study (αs = 0.69-0.79). Whereas the K-SADS-PL was used to assess categorical MDD diagnoses and timing of onset, the CDRS-R was used to assess continuous depressive symptom severity. Youth may exhibit elevated depressive symptoms without meeting full criteria for a depressive episode; therefore, including both approaches allowed us to examine both symptom trajectories and diagnostic onset.

Youth’s levels of co-rumination were assessed at baseline with the Co-Rumination Questionnaire (CRQ; Rose, 2002). The CRQ is a 27 item self-report measure that assesses the extent to which youths typically co-ruminate with their closest same-sex friend. The CRQ was originally developed and validated in the context of same-sex friendships in children and adolescents (Rose, 2002) based on data showing that during middle childhood and early adolescence, close friendships are typically same-sex (e.g., Kovacs et al., 1996), a finding that continues to be true in the decades since, with most discussions of stress continuing to occur with same-sex friends (Borowski & Rose, 2022). Assessing co-rumination in this context therefore aligns with both the original validation of the measure and developmental norms. Each item on the CRQ is rated on a 5-point Likert scale (i.e., 1 = Not at All True, 5 = Really True) and include statements such as “We spend most of our time together talking about problems that my friend or I have.” Items were averaged to create a mean co-rumination score, with higher scores reflecting greater co-rumination. The CRQ has demonstrated excellent reliability and validity in child and adolescent samples (Hankin et al., 2010; Rose, 2002, 2021; Rose et al., 2007; Stone et al., 2011) and strong retest reliability over six months (*r* = .54; Rose et al., 2007). The CRQ exhibited excellent internal consistency in this study (α = 0.97).

### Procedure

Participants were recruited from the community through various forms of advertisements (e.g., flyers and newspaper ads). During the baseline assessment, participating mothers provided informed consent and youth provided assent. After the consenting procedures, mothers completed the SCID-I while youth completed the CRQ, CDRS-R, and K-SADS in a separate room. Participants then completed follow-up assessments approximately every 6 months for two years (5 assessments total). In practice, the average duration between follow-ups was 6.17 months (*SD* = 0.94). During these follow-up assessments, youth were re-administered the CDRS-R and K-SADS to assess levels of depressive symptoms and onset of MDD episodes during the follow-up period. Families were compensated financially for participating in the study. All study procedures were approved by Binghamton University’s Institutional Review Board.

### Analysis Plan

We used hierarchical linear modeling (HLM 8; Raudenbush et al., 2019; Raudenbush & Bryk, 2002) to examine trajectories of offspring’s depressive symptoms (CDRS-R scores) across the two-year follow-up and to determine whether these trajectories were moderated by youth co-rumination across the follow-up period. The Level 1 (within subject) model was

$$\:CDRS-{R}_{ti}=\hspace{0.17em}{\pi\:}_{0i}\hspace{0.17em}+\hspace{0.17em}{\pi\:}_{1i}\:\left({Month}_{ti}\right)\:+\:{e}_{ti}$$

where *CDRS-R*_*ti*_ represents the CDRS-R score at month *i* for participant *j*, *π*_*0i*_ is the CDRS-R intercept (CDRS-R score at the initial assessment), *π*_*1i*_ is the slope of the linear change in CDRS-R scores over time (in months since the baseline assessment), and *e*_*ti*_ represents the error term. The Level 2 (between subjects) model was

$$\:\begin{array}{c}{\pi\:}_{0i}={{\upbeta\:}}_{00}\hspace{0.17em}+\hspace{0.17em}{{\upbeta\:}}_{01}\left(\mathrm{M}\mathrm{o}\mathrm{m}\:\mathrm{M}\mathrm{D}\mathrm{D}\right)+{{\upbeta\:}}_{02}(\mathrm{C}\mathrm{o}-\mathrm{r}\mathrm{u}\mathrm{m}\mathrm{i}\mathrm{n}\mathrm{a}\mathrm{t}\mathrm{i}\mathrm{o}\mathrm{n})+\\\:{{\upbeta\:}}_{03}(\mathrm{M}\mathrm{o}\mathrm{m}\:\mathrm{M}\mathrm{D}\mathrm{D}\times\:\mathrm{C}\mathrm{o}-\mathrm{r}\mathrm{u}\mathrm{m}\mathrm{i}\mathrm{n}\mathrm{a}\mathrm{t}\mathrm{i}\mathrm{o}\mathrm{n})+{r}_{0i}\end{array}$$

$$\:\begin{array}{c}{\pi\:}_{1i}={{\upbeta\:}}_{10}+{{\upbeta\:}}_{11}\left(\mathrm{M}\mathrm{o}\mathrm{m}\:\mathrm{M}\mathrm{D}\mathrm{D}\right)+{{\upbeta\:}}_{12}(\mathrm{C}\mathrm{o}-\mathrm{r}\mathrm{u}\mathrm{m}\mathrm{i}\mathrm{n}\mathrm{a}\mathrm{t}\mathrm{i}\mathrm{o}\mathrm{n})+\\\:{{\upbeta\:}}_{13}(\mathrm{M}\mathrm{o}\mathrm{m}\:\mathrm{M}\mathrm{D}\mathrm{D}\times\:\mathrm{C}\mathrm{o}-\mathrm{r}\mathrm{u}\mathrm{m}\mathrm{i}\mathrm{n}\mathrm{a}\mathrm{t}\mathrm{i}\mathrm{o}\mathrm{n})+{r}_{1i}\end{array}$$

This allowed us to examine the main and interactive effects of maternal history of MDD and children’s co-rumination (CRQ scores) on the CDRS-R intercept (*π*_*0i*_) reflecting levels of depressive symptoms at the initial assessment (month 0) and on trajectories of CDRS-R change over time (*π*_*1i*_) through month 24. Mother history of MDD (1 = yes, 0 = no) and co-rumination scores were grand centered before creating the interaction and entering the main effects and interaction into the model.

We then used Cox regression survival analyses in SPSS to examine risk for the onset of MDD in offspring during the two-year follow-up. These analyses focused on time to onset of youths’ first episode of MDD during the two-year follow-up. Using time to MDD diagnosis in youth as the outcome variable, we examined the main and interactive effects of mother MDD history and offspring co-rumination. As before, mother MDD and co-rumination scores were grand mean centered for this analysis. We accounted for right-censoring in our survival analysis models, such that youth who did not meet criteria for any episode of MDD by the end of the follow-up or who were lost to follow-up were treated as censored at their last available time point.

Finally, exploratory analyses were conducted to determine whether youth age or sex moderated any of the effects in the HLM models predicting depressive symptom trajectories or in the survival analyses predicting new onsets of MDD.

## Results

Of 214 the mother-offspring dyads participating in the initial assessment, 199, 189, 163, and 166 participated in the 6, 12, 18, and 24-month follow-ups, respectively, with 89.25% of dyads completing at least 3 of the 5 assessments. We examined whether participants who missed one or more assessments differ from those with complete data at all assessments on any demographic or clinical variables and, out of the 10 tests conducted, one was nominally significant. Specifically, in these analyses, children who missed at least one follow-up assessment had higher CDRS-R scores at T3 (*p* = .02) than children who completed all time points. We also examined Little’s Missing Completely at Random (MCAR) test (Little & Rubin, 1987), for which the null hypothesis is that the data are missing completely at random. This test was nonsignificant, χ^2^(105) = 104.03, *p* = .51, suggesting that the data were missing at random. We address missing data in our analyses in two ways. First, for the HLM models, we used full information maximum likelihood (FIML) estimates of missing data. This approach uses all available observed data to estimate model parameters and yields less biased and more efficient estimates than traditional approaches such as pairwise or listwise deletion (Schafer & Graham, 2002). For the survival analyses, if a family missed a follow-up assessment, the following K-SADS assessment focused on the entire time since the previous completed assessment. For example, if a family missed the 6-month assessment, the 12-month assessment focused on any episodes of MDD since Time 1 (T1). Of the 75 dyads who missed at least one follow-up assessment, 27 returned at a subsequent wave, and 17 only missed the final assessment. As noted above, participants who attritted from the study prior to the final follow-up assessment were treated as censored at their last available time point. Table 1 presents descriptive statistics for the two study groups.

**Table 1:** Descriptive statistics for study variables

	Offspring of Mothers with MDD	Offspring of Never Depressed Mothers
	(*n* = 106)	(*n* = 108)
Youth Sex (% girls)	47.20%	55.60%
Youth Race (% Non-Hispanic White)	74.50%	89.80%
Youth Age (T1)	11.59 (1.97)	11.43 (1.82)
CRQ (T1)	2.78 (1.01)	2.62 (0.92)
CDRS-R (T1)	22.90 (5.86)	19.28 (2.79)
CDRS-R (T2)	22.58 (6.48)	19.17 (2.77)
CDRS-R (T3)	21.85 (5.77)	19.44 (4.03)
CDRS-R (T4)	20.97 (4.89)	18.83 (2.87)
CDRS-R (T5)	20.82 (5.94)	19.44 (4.25)
Youth MDD during the follow-up (% yes)	10.38%	4.63%

Note: MDD = Major depressive disorder. CRQ = Co-Rumination Questionnaire. CDRS-R = Children’s Depression Rating Scale-Revised. Unless otherwise noted, values reflect means (standard deviations)

### Predicting Trajectories of Depressive Symptoms Over the Two-Year Follow-up

The results of these analyses are presented in Table 2. As can be seen in the table, mother MDD history significantly predicted youths’ levels of depressive symptoms at the baseline assessment (CDRS-R intercept), *t*(210) = 6.40, *p* < .001, *r*_*effect size*_ = 0.40,^2^ and linear change in depressive symptoms across the follow-up, *t*(210) = -2.73, *p* = .007, *r*_*effect size*_ = 0.19. In contrast, the main effect of co-rumination did not significantly predict the CDRS-R intercept, *t*(210) = 1.78, *p* = .08, *r*_*effect size*_ = 0.12, or change in CDRS-R scores across the follow-up, *t*(210) = -0.56, *p* = .58, *r*_*effect size*_ = 0.04. Importantly, the maternal MDD ⋅ co-rumination interaction significantly predicted children’s depressive symptom levels at the baseline assessment, *t*(210) = 2.73, *p* = .007, *r*_*effect size*_ = 0.19, but not change over time, *t*(210) = -0.32, *p* = .75, *r*_*effect size*_ = 0.02.^3^

**Table 2:** Summary of analyses predicting trajectories of depressive symptoms

Variable	b	t	*p*	*r*_effect size_
CDRS-R intercept (π_0_)				
Intercept (β_00_)	17.60	68.98	< 0.001	0.98
Mom MDD (β_01_)	3.28	6.40	< 0.001	0.40
CRQ (β_02_)	0.51	1.78	0.077	0.12
Mom MDD ⋅ CRQ (β_03_)	1.58	2.73	0.007	0.19
Month slope (π_1_)				
Intercept (β_10_)	-0.04	-2.62	0.009	0.18
Mom MDD (β_11_)	-0.08	-2.73	0.007	0.19
CRQ (β_12_)	-0.01	-0.56	0.576	0.04
Mom MDD ⋅ CRQ (β_13_)	-0.01	-0.32	0.749	0.02

Note: MDD = Major depressive disorder. CRQ = Co-Rumination Questionnaire

CDRS-R = Children’s Depression Rating Scale-Revised

**Fig. 1:**
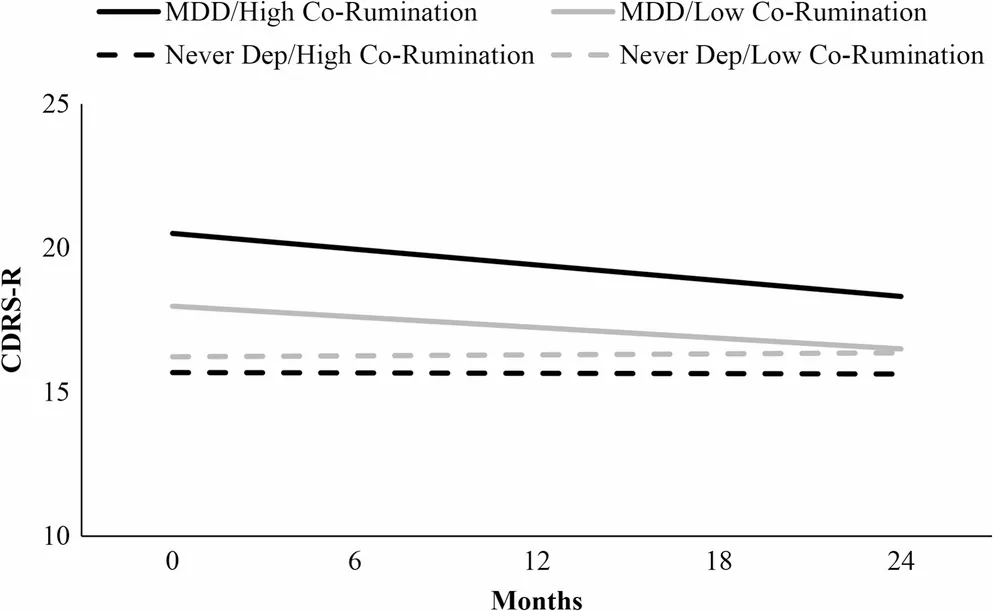
Trajectories of depressive symptoms over the two-year follow-up. *Note*: MDD = Major depressive disorder. CDRS-R = Children’s Depression Rating Scale-Revised

To determine the form of the interaction, we examined the main effect of co-rumination in each mother MDD group separately (see Fig. 1). Among offspring of mothers with MDD, levels of co-rumination predicted higher levels of depressive symptoms at the baseline assessment, *t*(104) = 2.38, *p* = .02, *r*_*effect size*_ = 0.23, but not change in depression over time, *t*(104) = -0.51, *p* = .61, *r*_*effect size*_ = 0.05. In contrast, among offspring of never depressed mothers, levels of co-rumination did not predict levels of depressive symptoms at baseline, *t*(106) = -1.42, *p* = .16, *r*_*effect size*_ = 0.14, or change in symptoms over time, *t*(106) = -0.29, *p* = .78, *r*_*effect size*_ = 0.03. These results indicate that, among offspring of mothers with MDD, but not among offspring of never depressed mothers, higher levels of co-rumination were associated with higher levels of depressive symptoms at the baseline assessment that appeared to be maintained across the full two years of the follow-up. To more formally evaluate whether baseline levels of co-rumination predicted higher levels of depressive symptoms to the end of the two-year follow-up among offspring of mothers with MDD, we conducted an additional analysis among offspring of mothers with MDD, re-centering the Months variable so that the zero point (intercept in the HLM analyses) reflected CDRS-R scores at the end of the follow-up (Month 24). In this analysis, the main effect of co-rumination remained significant, *t*(104) = 1.96, *p* = .05, *r*_*effect size*_ = 0.19. These results indicate that, among offspring of mothers with MDD, higher co-rumination levels predicted higher depressive symptoms at the baseline assessment and continued to predict higher depressive symptom levels at the end of the two-year follow-up, suggesting that higher levels of co-rumination were associated with chronic elevations of depressive symptoms in this group.

### Predicting New Onsets of MDD During the Follow-up

**Fig. 2:**
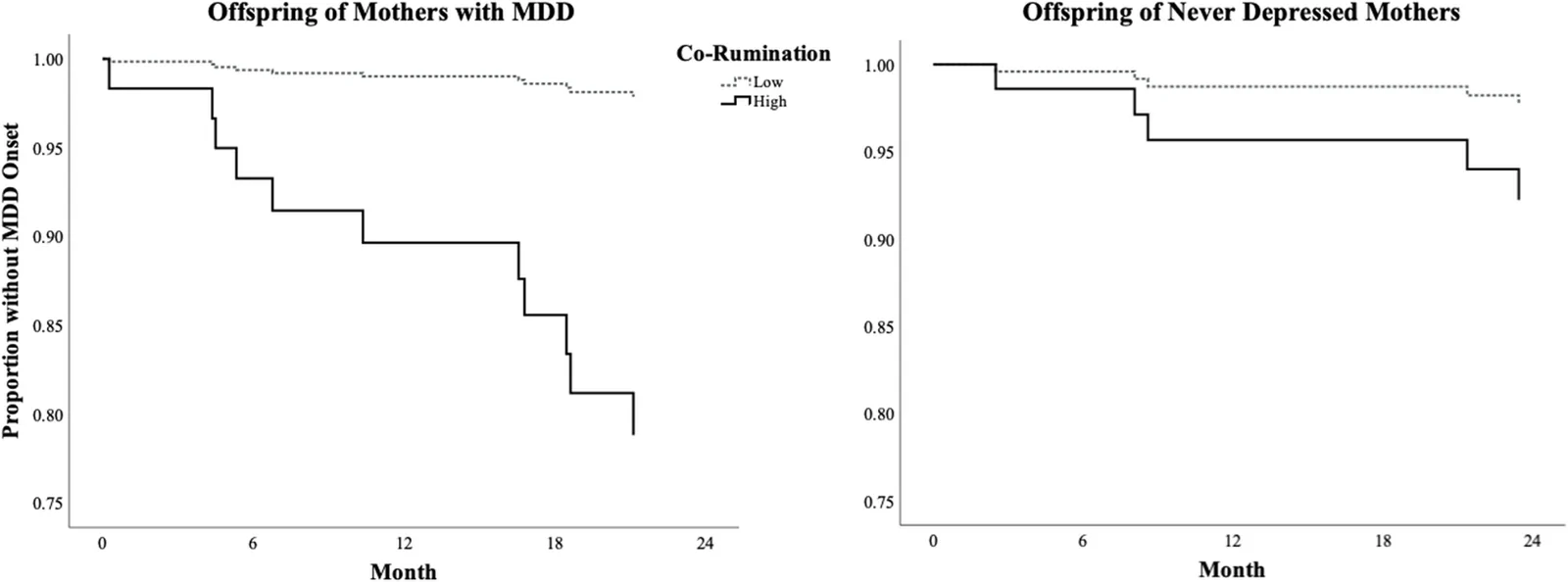
Summary of survival analyses predicting new onsets of major depressive disorder among offspring. *Note*: MDD = Major depressive disorder

We next used survival analysis to examine risk for the onset of MDD in youth during the two-year follow-up. As noted above, none of the youth met criteria for MDD at the baseline assessment. In this analysis, although the main effects of mother MDD, Wald = 2.10, *p* = .15, Hazard Ratio (HR) = 2.82, and youth co-rumination, Wald = 0.13, *p* = .72, HR = 0.89, in predicting the onset of MDD in youth were nonsignificant, there was a significant mother MDD ⋅ co-rumination interaction, Wald = 4.91, *p* = .03, HR = 4.31. To determine the form of this interaction, we conducted follow-up Cox regression analyses to examine the main effect of co-rumination in each mother MDD group separately. Higher levels of co-rumination predicted increased risk for MDD onset during the 2-year follow-up among offspring of mothers with MDD, Wald = 4.32, *p* = .04, HR = 1.85, but not among offspring of never depressed mothers, Wald = 2.01, *p* = .16, HR = 0.43. These findings are presented in Fig. 2 using a median split for CRQ scores to generate the figures.

Finally, exploratory analyses were conducted to determine whether youth age or sex moderated any of the effects in either the HLM models predicting depressive symptom trajectories or the survival analyses predicting new onsets of MDD episodes. None of these analyses was significant (lowest *p* = .15).

## Discussion

The goal of this study was to test a model of risk for depression in youth integrating family-based and peer-based influences. Consistent with our hypotheses, offspring of mothers with a history of MDD who also exhibit high levels of co-rumination with their peers were at greatest risk for depression during the two-year follow-up. Specifically, higher levels of co-rumination were associated with higher levels of depressive symptoms at the baseline assessment and throughout the end of the two-year follow-up among offspring of mothers with MDD, but not among offspring of never depressed mothers. These results suggest that higher levels of co-rumination were associated with consistently elevated levels of depressive symptoms in this high-risk group. In addition, higher levels of co-rumination predicted increased risk for the onset of MDD during the follow-up among offspring of mothers with MDD, but not among offspring of never depressed mothers.

These findings add to a growing body of research showing that peer-related influences can moderate risk for the intergenerational transmission of depression (for a review, see Goodman, 2020). Although co-rumination is often seen as a form of social support that strengthens perceived friendship quality (Rose, 2002, 2021; Rose et al., 2007), our findings suggest that it may have disproportionately negative effects for youth with a maternal history of MDD. One possible explanation for this pattern is that youth with a maternal history of MDD experience higher levels of chronic and episodic stress than offspring of never depressed mothers (Adrian & Hammen, 1993; Feurer et al., 2016), which in turn gives them more negative content to discuss with peers. Although social support is generally protective against depression in youth (Cairns et al., 2014; Rueger et al., 2016), co-rumination may function differently in this high-risk group, reinforcing maladaptive thinking patterns and coping strategies that contribute to the maintenance of depressive symptoms and increase the risk of depressive episodes (Badawi & Ingram, 2025; Hankin et al., 2010; Rose, 2002; Stone et al., 2011). The perceived social benefits of co-rumination may make it a particularly challenging risk factor to address since the sense of closeness it creates in friendships can reinforce ongoing negative self-disclosure, even when it worsens emotional distress (Hankin et al., 2010; Rose, 2002, 2021; Schwartz-Mette & Rose, 2012).

More broadly, these findings contribute to theoretical models of depression emphasizing the role of how interpersonal processes shape risk trajectories across development. Although offspring of mothers with MDD are exposed to elevated genetic and environmental vulnerability (Goodman, 2020), the current findings suggest that peer-related interpersonal processes amplify this risk, helping to explain why some high-risk youth develop depression whereas others do not. Specifically, prior research has shown that offspring of mothers with MDD experience higher levels of stressful events, particularly interpersonal stressors, than offspring of never depressed mothers (Adrian & Hammen, 1993; Feurer et al., 2016). Research has also shown that co-rumination can increase the salience of negative experiences with others, reinforcing maladaptive emotional responses to stress, and contributing to cycles of stress generation and depression risk (Rose et al., 2014, 2017; Bastin et al., 2015; Huang et al., 2022). The current findings, therefore, help to integrate family history and interpersonal models of depression risk by demonstrating how co-rumination with peers may be a particularly strong risk factor among youth already at high risk for depression based on a maternal history of MDD.

This said, the current findings do not establish co-rumination as a causal mechanism underlying depression risk, which would require an examination of how changes in co-rumination lead to increases or decreases on depression, but instead suggest that co-rumination may serve as an important marker for identifying youth at elevated risk, particularly among offspring of mothers with MDD. Although co-rumination can feel socially rewarding, prior work suggests that it may exacerbate rather than mitigate risk. Future prevention and intervention studies are needed to determine whether modifying co-rumination leads to subsequent reductions in depression risk. Notably, emerging evidence suggests that youth with higher levels of co-rumination may benefit from rumination-focused interventions (Kaufman et al., 2025), and prevention programs specifically targeting co-rumination are beginning to be developed (Vuijk et al., 2024). Thus, although evidence supporting co-rumination as a modifiable mechanism remains limited, developing effective ways of reducing co-rumination among at-risk youth may represent a promising avenue for intervention. Programs focusing on enhancing adaptive communication, interpersonal skills, and problem-solving in peer relationships could help high-risk youth seek support in healthier ways without reinforcing depressive thought patterns (e.g., Metz et al., 2023; Young et al., 2016; Zheng et al., 2023). Additionally, interventions designed to build resilience in youth may help them develop healthier coping strategies and reduce reliance on co-rumination as a means of managing stress (e.g., Kallianta et al., 2021; Noyola et al., 2024). These types of interventions may be particularly important for youth already at heightened risk for depression, such as offspring of mothers with MDD.

The current study has several strengths including the multi-wave prospective design and use of diagnostic interviews, which allowed us to examine for the first time whether co-rumination may amplify depression risk among offspring already at elevated intergenerational risk due to maternal MDD history. In addition, the study expands upon prior research by demonstrating that co-rumination may be particularly deleterious in youth already at heightened risk due to maternal MDD history. However, this study also had limitations, which provide important future directions of research. First, although the number of depression diagnoses in offspring during the follow up was sufficient for our primary analysis, the relatively small number of depressive onsets may have limited statistical power, and the precision of interaction estimates in the survival analyses. Therefore, replication in larger samples with greater numbers of depressive onsets is needed to evaluate the robustness of these findings, particularly for interaction effects. In addition, our exploratory analyses to examine age and sex as potential moderators were underpowered. Therefore, future research with larger samples is needed to determine whether the effects of co-rumination on depression risk differ by age or sex as presented by prior research (Rose, 2002). A second limitation was that our sample was mostly non-Hispanic White, which limits the generalizability of our findings to more racially and ethnically diverse populations. Finally, we used the CRQ to assess levels of co-rumination, which has been used successfully for over 20 years, and which demonstrated strong predictive validity in our study. However, newer approaches have been developed that may allow a more flexible and fine-grained assessment of co-rumination among peers including behavioral coding of co-rumination within peer dyad discussions (e.g., Rose et al., 2005) and methods of coding co-rumination that may occur via social media (e.g., Barreira et al., 2025).

Notably, the current results suggest that the effects of co-rumination may differ based on risk level, with high-risk youth (e.g., those with maternal MDD) experiencing more negative consequences and fewer social benefits, whereas low-risk youth may derive more positive effects with less emotional costs. Future research should examine perceived friendship quality to better understand how the interplay of these positive and negative aspects of co-rumination varies across risk levels. Moreover, although our study investigated co-rumination with same-sex peers, co-rumination has also been observed within parent–adolescent relationships, and research indicates that youth with depression engage in elevated co-rumination with both peers and parents (Grimbos et al., 2013; Ioffe et al., 2020; Waller et al., 2014). These findings suggest that co-rumination may reflect a broader interpersonal communication style that generalizes across close relationships rather than being limited to peer interactions. Future research should examine whether youths’ tendencies to co-ruminate across multiple interpersonal contexts confer additive or interactive risk for depression. Incorporating these approaches within multi-wave longitudinal studies would help clarify the role of co-rumination across interpersonal contexts in the intergenerational transmission of depression. Lastly, because the current study focused specifically on maternal history of MDD, future research should examine whether similar patterns emerge for paternal depression or depression in other caregivers to better understand how different family influences interact with peer processes in shaping youth depression risk.

In summary, the results of this study support the hypothesis that co-rumination with peers predicts depression risk among offspring of mothers with MDD. Specifically, among offspring of mothers with a history of MDD, higher levels of co-rumination predicted higher levels of depressive symptoms at baseline and throughout a two- year-follow-up, as well as increased risk for MDD onset. Because co-rumination represents a malleable risk factor, it may provide an important target for interventions designed to reduce risk for depression in youth.

## Data Availability

Data reported in this article are available upon request from the corresponding author.

## Footnotes

1 The only difference in diagnostic criteria between DSM-IV and DSM-5 is that DSM-5 removed the bereavement exclusion, allowing diagnoses of MDD within two months of the death of a loved one without requiring extra symptoms or impairment. Therefore, everyone who met DSM-IV criteria for MDD in our study would also meet DSM-5 criteria for MDD.

2 Effect sizes were expressed as *r*_*effect size*_, calculated from the t statistic using the formula *r* = √(*t*^*2*^ */ (t*^*2*^ *+ df)* (Rosenthal & Rosnow, 1991), with values of 0.10, 0.30, and 0.50 reflecting small, medium, and large effects, respectively.

3 Exploratory analyses tested a quadratic model of change in symptoms across the follow-up by adding Month ⋅ Month term to the Level 1 model. Adding this quadratic term did not improve model fit and none of the main or interaction effects of the quadratic term were significant. Details of this analysis are available from the first author.
